# Is There an Increased Risk of Post-Operative Surgical Site Infection after Orthopaedic Surgery in HIV Patients? A Systematic Review and Meta-Analysis

**DOI:** 10.1371/journal.pone.0042254

**Published:** 2012-08-08

**Authors:** James W. M. Kigera, Masja Straetemans, Simplice K. Vuhaka, Ingeborg M. Nagel, Edward K. Naddumba, Kimberly Boer

**Affiliations:** 1 Department of Orthopaedics, College of Health Sciences, Makerere University, Kampala, Uganda; 2 Orthopaedic Rehabilitation Centre, PCEA Kikuyu Hospital, Kikuyu, Kenya; 3 Department of Biomedical Research, Royal Tropical Institute (KIT), Amsterdam, The Netherlands; 4 Department of Information and Library Services, Royal Tropical Institute (KIT), Amsterdam, The Netherlands; 5 Department of Orthopaedics, Mulago National Referral Hospital, Kampala, Uganda; 6 Academic Medical Centre, Centre for Poverty-Related Communicable Disease AMC-CPCD/AIGHD, Kigali, Rwanda and Amsterdam, The Netherlands; University of Cape Town, South Africa

## Abstract

**Background:**

There is dilemma as to whether patients infected with the Human Immunodeficiency Virus (HIV) requiring implant orthopaedic surgery are at an increased risk for post-operative surgical site infection (SSI). We conducted a systematic review to determine the effect of HIV on the risk of post-operative SSI and sought to determine if this risk is altered by antibiotic use beyond 24 hours.

**Methods:**

We searched electronic databases, manually searched citations from relevant articles, and reviewed conference proceedings. The risk of postoperative SSI was pooled using Mantel-Haenszel method.

**Results:**

We identified 18 cohort studies with 16 mainly small studies, addressing the subject. The pooled risk ratio of infection in the HIV patients when compared to non-HIV patients was 1.8 (95% Confidence Interval [CI] 1.3–2.4), in studies in Africa this was 2.3 (95% CI 1.5–3.5). In a sensitivity analysis the risk ratio was reduced to 1.4 (95% CI 0.5–3.8). The risk ratio of infection in patients receiving prolonged antibiotics compared to patients receiving antibiotics for up to 24 hours was 0.7 (95% CI 0.1–4.2).

**Conclusions:**

The results may indicate an increased risk in HIV infected patients but these results are not robust and inconclusive after conducting the sensitivity analysis removing poor quality studies. There is need for larger good quality studies to provide conclusive evidence. To better develop surgical protocols, further studies should determine the effect of reduced CD4 counts, viral load suppression and prolonged antibiotics on the risk for infection.

## Introduction

In Sub-Saharan Africa, the orthopaedic surgeon is handling increasing numbers of trauma cases due to increasing road traffic accidents [1]–[3]. Additionally the African orthopaedic surgeon is faced with the HIV/AIDS epidemic, with increasing numbers of HIV infected patients, many of whom do not yet show symptoms, and have not yet started antiretroviral therapy (ART) [4]. The prevalence of HIV in the general population in Sub-Saharan Africa ranges from 3%–12% [5], while the prevalence of HIV infection among patients requiring orthopedic surgery ranges from 3.6% to 16% [6], [4]. The higher rates of 16% seen in Africa are probably due to the large numbers of young people vulnerable to trauma after road traffic accidents. This age group also has a higher HIV prevalence.

Surgery is considered clean if it is conducted in uncontaminated or uninfected tissues and the respiratory, gastrointestinal and genitourinary systems are not opened [7]. Without concomitant disease, such as HIV, surgical operations have less than 2% risk for post operative surgical site infections [8]–[10]. It has been postulated that in patients infected with HIV, the risk of postoperative infection is increased due to the decline in the number of CD4 cells [11]. Untreated HIV causes a gradual decline in CD4 counts with subsequent increase in opportunistic infections. It may also lead to an increase in the incidence of infection after surgery. It is expected that the risk reduces once the patient is on ART and the CD4 counts rise. Surgery in orthopaedics sometimes requires the insertion of implants of various biomaterials to replace a joint surface or to stabilise bone fragments. The use of implants is associated with an increase in the risk of postoperative infection [10]. Because a foreign body is implanted in the body which provides an area for possible colonisation by microbes, there is not only an increase risk of infections occurring in the first one month (early infection) following surgery, but also up to one year postoperatively (late infection). Infected implants are usually managed by antibiotics for long durations and removal or exchange of the implant all resulting in great morbidity and cost [12], [13].

Presently there is conflicting data on whether HIV or reduced CD4 count due to HIV increases the likelihood of infections in clean implant surgery [14]–[17], [7]. The dilemma about not knowing whether implant surgery is safe for HIV positive individuals, has led surgeons to believe that the risk of infection in HIV infected patients is too high. They avoid elective surgery and only consider emergency surgery [18]. This means that, with one in every six patients requiring orthopedic surgery being infected with HIV, denying this group of patients elective surgery leaves a large number of HIV infected patients who may be denied surgery based on an unsubstantiated risk of increased infection leading to reduced quality of life for these patients. With most large hospitals in East Africa performing about 7 implant orthopaedic surgeries a day, this could mean that about 300 patients a year in each of these hospitals may be denied elective surgery and suffer reduced quality of life [4], [19].

According to several American and European guidelines, prophylactic antibiotics should be started within one hour of the incision and stopped within 24 hours after the end of the operation [20]–[22]. By following these current protocols for implant surgery, the risk of post-operative infection has been greatly reduced [23]. In clean implant orthopedic surgery we can expect an infection rate of less than 2% [8], [9].

Though there are guidelines on the perisurgical management of patients undergoing implant surgery, none specifically address the HIV infected patient. Therefore there is need to develop guidelines for the orthopedic surgeon working in areas of high prevalence of HIV. Our study aims to gather the best evidence available on the risk of infection after clean implant orthopedic surgery in patients with HIV compared to patients without HIV to support the development of these guidelines.

We have conducted a systematic literature review to determine firstly, the incidence of post-operative surgical site infections in patients with HIV undergoing clean implant orthopedic surgery compared to patients without HIV. Secondly, we identified studies that evaluated the effect of the enhanced measure of prolonged antibiotic use compared to antibiotics given for up to 24 hours (standard care in most countries) in reducing the risk of post-operative infection in HIV infected patients.

## Materials and Methods

A protocol was developed in advance of conducting this systematic review and meta-analysis following the Cochrane Collaboration protocol development guidelines [24].

To identify studies assessing the incidence of early post-operative infection in clean implant orthopaedic surgery patients with HIV compared to those without HIV, we searched for publications in the Pubmed, Embase and CENTRAL databases in June 2012 without restrictions on year of publication. The combination of key words (exploded MESH headings and free text terms) in the search strategy included HIV/AIDS, implant orthopaedic surgery, post-operative complications and surgical site infections (Table 1). Furthermore, the reference lists of eligible studies were searched for any additional studies. We also searched abstracts of relevant Orthopaedic and HIV/AIDS conferences (by searching the Journal of Bone and Joint Surgery database of conference proceedings and the International AIDS Society website) in June 2012 without restrictions on year of publication. We contacted authors of eligible studies to identify additional published and unpublished studies.

**Table 1:** SEARCH STRATEGY PUBMED.

(“HIV Infections”[Mesh] OR “HIV”[Mesh] OR “Acquired Immunodeficiency Syndrome”[Mesh] OR HIV[tiab] OR HIV-1[tiab] OR HIV-2[tiab] OR human immunodeficiency virus[tiab] OR human immunedeficiency virus[tiab] OR human immuno-deficiency virus[tiab] OR human immune-deficiency virus[tiab] OR (human immun*[tiab] AND deficiency virus[tiab]) OR acquired immunodeficiency syndrome[tiab] OR acquired immuno-deficiency syndrome[tiab] OR acquired immune-deficiency syndrome[tiab] OR (acquired immun*[tiab] AND deficiency syndrome[tiab])) AND (“Orthopedics”[Mesh] OR “Orthopedic Procedures”[Mesh] OR “Joint Prosthesis”[Mesh] OR “Fracture Fixation, Internal”[Mesh] OR “Orthopedic Fixation Devices”[Mesh] OR “Arthroplasty”[Mesh] OR orthopedic*[tiab] OR orthopaedic*[tiab] OR prosthes*[tiab] OR prosthetic[tiab] OR (implant*[tiab] AND (joint[tiab] OR elbow[tiab] OR knee[tiab] OR hip[tiab] OR bone[tiab])) OR fracture fixat*[tiab] OR internal fixat*[tiab] OR osteosynthes*[tiab] OR arthroplast*[tiab]) AND (“Wound Infection”[Mesh] OR “Prosthesis-Related Infections”[Mesh] OR “Soft Tissue Infections”[Mesh] OR “Surgical Wound Dehiscence”[Mesh] OR wound[tiab] OR wounds[tiab] OR infection*[tiab] OR infected[tiab])

Our first question concentrated on the incidence of post operative surgical site infection after clean implant orthopaedic surgery in HIV infected patients compared to non-HIV infected patients. Eligible studies were retrospective and prospective cohort studies that had one group of HIV infected patients and another group of non-HIV infected patients; we included studies in which there were no patients operated while having an infection at time of surgery, hence all occurrences of infections could be considered as incident events. All participants underwent clean implant orthopaedic surgery and the incidence of post operative surgical site infection was evaluated. Surgery is considered clean if it was conducted in uncontaminated or uninfected tissues and the respiratory, gastrointestinal and genitourinary systems are not opened (13).

The second question was on the effect of prolonged antibiotics on the incidence of post-operative infection in HIV infected patients after clean implant orthopaedic surgery. The eligible studies were randomized control trials, quasi-randomized control trials and cohort studies comparing infection rates among HIV patients receiving antibiotics for up to 24 hours and those receiving antibiotics for longer than 24 hours.

For both questions, studies published in English, French, Dutch or German were included. Studies on patients with open fractures or surgery done in the presence of infection were excluded.

**Figure 1:**
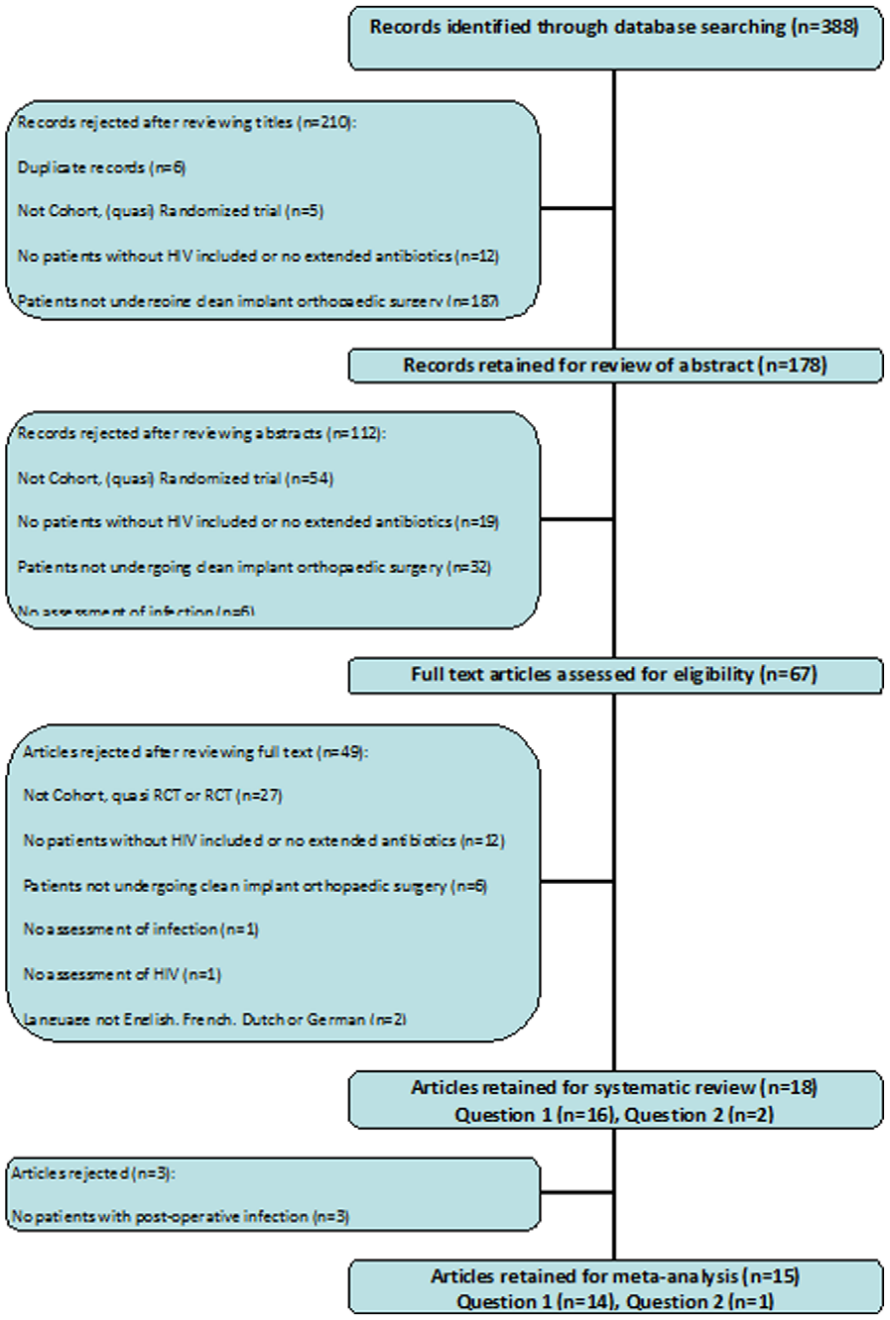
Flow chart of selection of studies. The figures indicate the number of articles reviewed at each stage.

Identified studies were reviewed for eligibility by two authors (JK and SV) based first on the title, then the abstract and then finally on the full text (Figure 1), disagreements were resolved by consensus and if none was arrived at, by discussion with a third author (MS/KB). Studies with data on infection rates and those with at least one case of infection identified were selected for the meta-analysis. Data extraction was completed by two authors (JK and SV) independently using a pre-designed data extraction form. We abstracted data on average age, sex, method of diagnosing HIV status, patient numbers, antibiotics used, methods of assessing infection and the number of patients who developed post operative surgical site infections. Although we originally planned to abstract data on number of episodes of infection this information was not reported. Disagreements on data extraction were resolved by consensus and if none was arrived at, by discussion with a third author (MS/KB).

### Quality Assessment

The studies identified were assessed for the quality of the study. Cohort studies were assessed using the Newcastle-Ottawa scale [25] while randomised and quasi randomised studies were assessed using the PEDro critical appraisal tool [26]. The Newcastle-Ottawa scale has all the important components for assessment of quality for cohort studies and was deemed appropriate for this study. The PEDro tool was developed using the Delphi consensus for quality assessment of RCTs and is appropriate for this setting. The assessment of quality was done by two authors (JK and SV) and disputes resolved by consensus and if none was arrived at, by discussion with a third author (MS/KB).

### Data Synthesis

The risk ratios estimating the risk of infection in the HIV patients compared to the infection in the non-HIV patients of the individual studies were combined using the Mantel-Haenszel method. Heterogeneity across studies was assessed firstly by eye-balling, followed by using the I^2^ and the Chi Square tests. Should the p-value of the heterogeneity test be <0.05, we planned to use the Random Effects Model (REM) instead of Fixed Effects Models (FEM). In the pre-specified subgroup analyses we estimated the risk ratios of post operative infection in the following populations:

- Studies conducted in the African continent which is the main area of interest because it is the area of the world with the highest prevalence and incidence of HIV [27]
- Studies done among patients suffering from haemophilia because this is the commonest co-morbidity among HIV patients in Europe and North America and these patients are different from non haemophilic patients [28]
- Studies showing infection in the first 30 days after surgery because we wanted to assess whether there is a difference in the risk for post operative surgical site infection in the early phase (within 30 days) when compared to the late phase (after 30 days)

Sensitivity analysis was conducted by excluding studies with lower quality as assessed by the quality tool. Publication bias was assessed using a funnel plot. Analysis was conducted using Revman version 5.

## Results

We retrieved 388 articles after a search of Pubmed, Embase and CENTRAL databases. Two hundred and ten articles were rejected after reading through title only and a further 112 after reviewing the abstracts. An additional 49 articles were excluded after reading through the full text; with the frequent reason for rejection being due to study designs not being cohorts, RCTs or quasi RCTs. The other common reason for rejection was the lack of non-HIV controls to answer the first research question or the lack of control patients on up to 24 hours of antibiotics to answer the second question (Figure 1).

We identified 18 studies that fulfilled the eligibility criteria for the systematic review sixteen for question 1 and two studies for question 2. All were cohort studies with four conducted in Africa and the rest in Europe and North America. The studies were mainly small with majority having less than 100 patients. The total number of patients with HIV was 402 and there were 1064 non-HIV infected patients. There was only one study available that was conducted outside of Africa which did not concentrate on patients with haemophilia. The characteristics of the studies included in the review are detailed in Table 2.

**Table 2:** Table showing the characteristics of studies included and the data abstracted

1^st^ Author, Publication Year	Country Conducted	Type of Patients	Average Age (Years)	Sex – Male/Female	Antibiotic Used	Mean Follow up	% of Patients with Infections in HIV group	% of Patients with Infections in Non HIV group
Chapman,2003 [33]	England	Haemophilia	48.4	5/0	Cefuroxime1.5 g Stat, 8 hrs, 16 hrs	42 Months	100%	0%
Goddard,2010 [34]	United Kingdom	Haemophilia	43	57/0	3^rd^ Generation Cephalosporin 1 dose pre-op, 2 doses post-p	9.2 Years	6.3%	0%
Harrison, 2002 [35]	Malawi	General Population	?	?	Cefazolin 1 g Stat	3 months	3.6%	5.6%
Hoekman, 1991 [31]	Rwanda	General Population	?	169/45	None	30 months	9.3%	4.7%
Jellis 1996 [30]	Zambia	General Population	?	?	?	?	32.1%	12.3%
Kelley,1995* [36]	United States	Haemophilia	38	14/0	?	8 years	0%	0%
Lehman,2001 [37]	United States	Haemophilia	?	?	?	At least 2 years	26.1%	33.3%
Lofquist,1996 [38]	Sweden	Haemophilia	46	11/0	?	?	50%	0%
Lubega,2010* [39]	Malawi	General Population	52	33/25	Cefuroxime 1500 mg Stat then 750 mg 3 doses	?	0%	0%
Norian,2002 [40]	United States	Haemophilia	33.7	41/0	?	Minimum 2 years	13.8%	25%
Paiement,1994 [41]	United States	General Population	?	?	?	Average 26 weeks	0%	4.3%
Powell,2005 [42]	United States	Haemophilia	32.5	32/0	?	Median 80 months	15.8%	15.4%
Rodriguez,2007 [43]	Spain	Haemophilia	31	30/0	? Drug For Two days	Average 7.5 Years	5.3%	0%
Silva,2005 [44]	United States	Haemophilia	40.1	87/0	? Drug for 3–5 Days	Average 7.8 Years	16.7%	13.3%
Solimeno,2009 [45]	Italy	Haemophilia	39	92/0	Cefotaxime 2 g and teicoplanin 400 mg	Median 5.1 Years	9.1%	10.2%
Vastel,1999 [46]	France	Haemophilia	40.8	21/0	?	Average 4.8 Years	41.7%	11.1%
Unger 1995* [16]	United States	Haemophilia	33	15/0	?	Average 6.4 Years	0%**	0%***
Bahebeck 2009 [29]	Cameroon	General Population	39	440/206	Cefuroxime 1.5 g stat/Cefuroxime 750 mg bd 10 days	At least 3 months	6.7%**	4.5%***

* = Excluded from meta-analysis

** = Study answering question two, group with standard of care

*** = Study answering question two, group with enhanced measure

? = Value of Item unclear from study

% = Percentage

In 10 studies the CD4 counts were available for at least some of the patients, only two studies reported on the use of ARVs among the patients and one reported on viral loads of the patients

**Figure 2:**
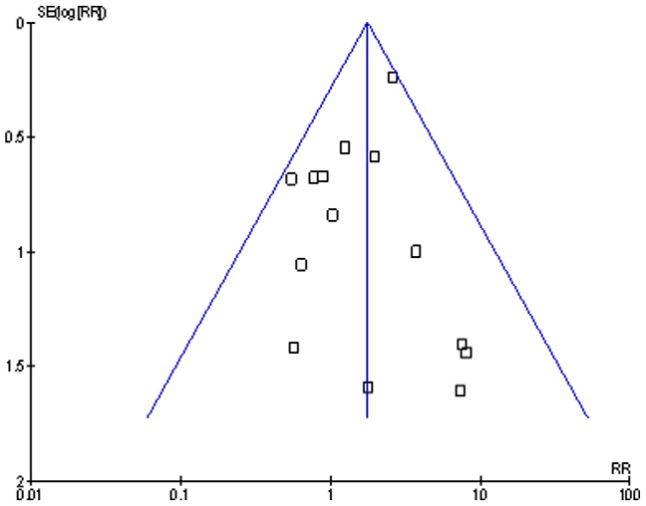
Funnel plot of studies estimating the risk ratio of post operative surgical site infections after clean orthopaedic implant surgery in HIV infected patients compared to HIV negative patients. Points indicate the relative risks (x-axis) from 14 studies assessing the risk of post operative surgical site infections after implant orthopaedic surgery in HIV infected patients when compared to HIV negative patients.

The quality of the studies as assessed using the Newcastle-Ottawa scale is shown in Table 3 and Table 4. On average the quality of the studies scored 81%. The areas that scored poorest were in the methods of ascertaining the HIV status (exposure) and determining post-operative surgical site infection (outcome). The representativeness of the cohorts to the population and the comparability of the cohorts scored above 90%. The risk of publication bias is shown in the funnel plot (Figure 2).

**Table 3:** Table showing the quality of studies comparing HIV infected and Non HIV infected cohorts.

Author, publication year	Representativeness of Exposed Cohort (HIV)	Selection of Non Exposed Cohort (Non HIV)	Ascertainment of Exposure (HIV)	Outcome not present at Start	Comparability of Cohorts	Assessment of Outcome (Infection)	Length of Follow-up	% of Follow-up	Score
Chapman,2003 [33]	Y	Y	?	Y	Y	?	Y	Y	75%
Goddard,2010 [34]	Y	Y	Y	Y	Y	?	Y	Y	87.5%
Harrison, 2002 [35]	Y	Y	Y	Y	Y	Y	Y	Y	100%
Hoekman, 1991 [31]	Y	Y	Y	Y	Y	Y	Y	Y	100%
Jellis 1996 [30]	Y	Y	?	Y	Y	Y	?	?	62.5%
Kelley,1995 [36]	Y	Y	?	Y	Y	?	Y	Y	75%
Lehman,2001 [37]	Y	N	Y	Y	Y	?	Y	Y	75%
Lofquist,1996 [38]	Y	Y	?	Y	Y	?	Y	Y	75%
Lubega,2010 [39]	Y	Y	Y	Y	Y	?	?	?	62.5%
Norian,2002 [40]	Y	Y	Y	Y	Y	?	Y	Y	87.5%
Paiement,1994 [41]	Y	Y	Y	Y	Y	Y	Y	Y	100%
Powell,2005 [42]	Y	Y	N	Y	Y	Y	Y	N	75%
Rodriguez,2007 [43]	Y	Y	?	Y	Y	?	Y	Y	75%
Silva,2005 [44]	Y	Y	N	Y	Y	Y	Y	N	75%
Solimeno,2009 [45]	Y	Y	?	Y	Y	?	Y	Y	75%
Vastel,1999 [46]	Y	Y	?	Y	Y	?	Y	Y	75%

Y = Item Catered for in Study

? = Unclear if Item is catered for

N = Item not catered for

**Table 4:** Table showing the quality of studies comparing Antiretroviral Drugs/Extended Antibiotics and No Antiretroviral Drugs/Standard Antibiotics cohorts

Papers	Representativeness of Exposed Cohort (ARV/Antibiotics)	Selection of Non Exposed Cohort (HIV)	Ascertainment of Exposure (ARV/Antibiotics)	Outcome not present at Start	Comparability of Cohorts	Assessment of Outcome (Infection)	Length of Follow-up	% of Follow-up	Score
Bahebeck, 2009 [29]	Y	Y	Y	Y	Y	Y	Y	Y	100%
Unger, 1995 [16]	Y	?	Y	Y	Y	?	Y	Y	75%

Y = Item Catered for in Study

? = Unclear if Item is catered for

N = Item not catered for

### Risk of Postoperative Infection after Implant Surgery

**Figure 3:**
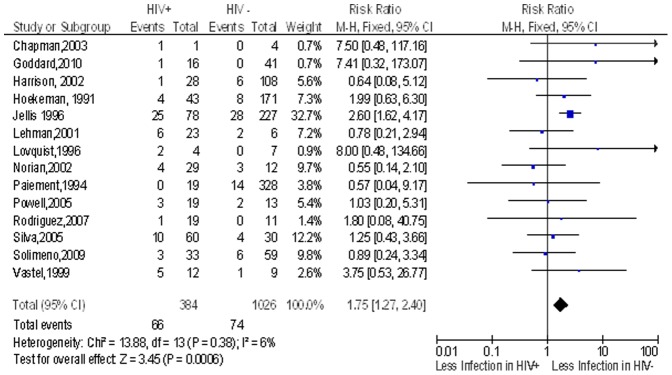
Risk of Infection after Implant Surgery in HIV patients compared to non HIV patients. Study or Subgroup on the Y-axis refers to first author and publication year; events refers to the number of patients who suffered post operative surgical site infections while total refers to the number of patients in that group. Weight refers to influence of each study on overall estimate (weights are from fixed effect analyses); for each study the central square indicates risk ratio, line represents 95% confidence interval (CI), and the size of the square reflects the study's weight in the pooling; overall estimate refers to pooled estimate of risk ratio after mathematical combination of all studies; the X-axis indicates the scale and the direction of the effect of HIV status on the risk of post operative surgical site infection. I-squared denotes the extent of heterogeneity in study outcomes, with a (hypothetical) value of 100% meaning considerable heterogeneity and 0% meaning no heterogeneity between studies.

We identified 16 studies that determined the risk of post-operative infection after clean implant orthopaedic surgery in the HIV infected patients compared to non-HIV patients but only 14 studies had patients that developed post-operative infection. The overall pooled proportion of individuals with post operative surgical site infection in the non HIV group was 7.2% while in the HIV infected group 17.2% of the individuals had post operative surgical infections. HIV infected individuals had an almost two times more risk (n = 66/384) of post-operative surgical site infection in the compared to individuals without HIV (n = 74/1026) with a risk ratio (RR) of 1.8 (95% CI 1.3–2.4) (Figure 3).

**Figure 4:**
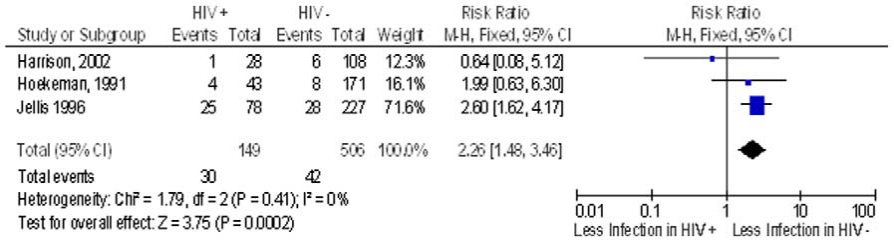
Overall infections in patients undergoing surgery in the African continent. Study or Subgroup on the Y-axis refers to first author and publication year; events refers to the number of patients who suffered post operative surgical site infections while total refers to the number of patients in that group. Weight refers to influence of each study on overall estimate (weights are from fixed effect analyses); for each study the central square indicates risk ratio, line represents 95% confidence interval (CI), and the size of the square reflects the study's weight in the pooling; overall estimate refers to pooled estimate of risk ratio after mathematical combination of all studies; the X-axis indicates the scale and the direction of the effect of HIV status on the risk of post operative surgical site infection. I-squared denotes the extent of heterogeneity in study outcomes, with a (hypothetical) value of 100% meaning considerable heterogeneity and 0% meaning no heterogeneity between studies.

**Figure 5:**
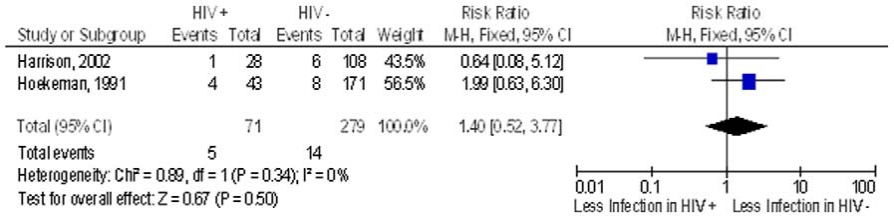
Risk of infection in patients undergoing surgery in the African continent after removing poorer quality studies. Study or Subgroup on the Y-axis refers to first author and publication year; events refers to the number of patients who suffered post operative surgical site infections while total refers to the number of patients in that group. Weight refers to influence of each study on overall estimate (weights are from fixed effect analyses); for each study the central square indicates risk ratio, line represents 95% confidence interval (CI), and the size of the square reflects the study's weight in the pooling; overall estimate refers to pooled estimate of risk ratio after mathematical combination of all studies; the X-axis indicates the scale and the direction of the effect of HIV status on the risk of post operative surgical site infection. I-squared denotes the extent of heterogeneity in study outcomes, with a (hypothetical) value of 100% meaning considerable heterogeneity and 0% meaning no heterogeneity between studies.

In a subgroup analysis, we reviewed the studies done in Africa (n = 4). Of these four studies, only three had the outcome of post-operative surgical site infection (both early and late), the pooled proportion of post operative surgical site infection after clean implant orthopaedic surgery in the non HIV group was 8.3% while in the HIV infected group this was 20.1%. The overall RR of post-operative infection after clean implant orthopaedic surgery in the HIV infected patients compared to non-HIV patients was 2.3 (95% CI 1.5–3.5; n = 655) (Figure 4). In a sensitivity analysis conducted removing one lower quality study that did not include ascertainment of exposure to HIV and had no follow up information, the risk ratio fell to 1.4 (95% CI 0.5–3.8; n = 350) (Figure 5).

**Figure 6:**
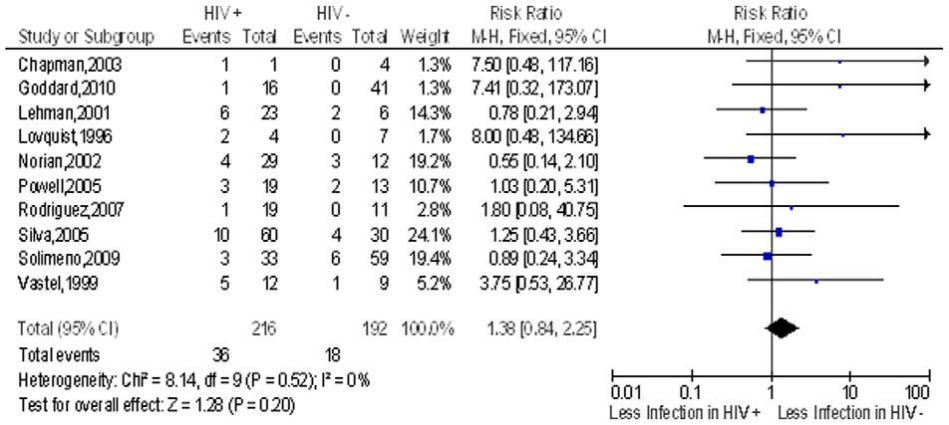
Overall risk of Infection in patients suffering from haemophilia. Study or Subgroup on the Y-axis refers to first author and publication year; events refers to the number of patients who suffered post operative surgical site infections while total refers to the number of patients in that group. Weight refers to influence of each study on overall estimate (weights are from fixed effect analyses); for each study the central square indicates risk ratio, line represents 95% confidence interval (CI), and the size of the square reflects the study's weight in the pooling; overall estimate refers to pooled estimate of risk ratio after mathematical combination of all studies; the X-axis indicates the scale and the direction of the effect of HIV status on the risk of post operative surgical site infection. I-squared denotes the extent of heterogeneity in study outcomes, with a (hypothetical) value of 100% meaning considerable heterogeneity and 0% meaning no heterogeneity between studies.

There were 11 studies done on patients with haemophilia with 10 studies having patients with the outcome of post-operative infection (both early and late); the proportion of post-operative infection in the non-HIV group was 9.4% while that in the HIV infected group was 16.7%. The overall RR of post-operative infection in the HIV infected patients when compared to non HIV patients in this haemophilia sub group was 1.4 (95% CI 0.8–2.3; n = 408) (Figure 6).

**Figure 7:**
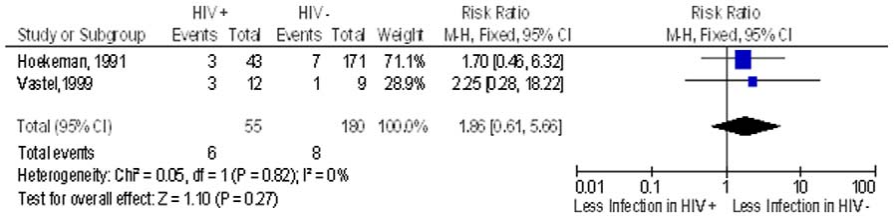
Risk of Infection in the first 30 days post operatively. Study or Subgroup on the Y-axis refers to first author and publication year; events refers to the number of patients who suffered post operative surgical site infections while total refers to the number of patients in that group. Weight refers to influence of each study on overall estimate (weights are from fixed effect analyses); for each study the central square indicates risk ratio, line represents 95% confidence interval (CI), and the size of the square reflects the study's weight in the pooling; overall estimate refers to pooled estimate of risk ratio after mathematical combination of all studies; the X-axis indicates the scale and the direction of the effect of HIV status on the risk of post operative surgical site infection. I-squared denotes the extent of heterogeneity in study outcomes, with a (hypothetical) value of 100% meaning considerable heterogeneity and 0% meaning no heterogeneity between studies.

Data for post-operative infection in the early postoperative period was only presented in two of the eleven studies; in these studies HIV infected patients showed an increased risk of developing post-operative infections compared to non-HIV patients, however it was not significant (RR:1.8; 95% CI 0.6–5.6; n = 235) (Figure 7).

In all the fourteen (14) studies included, there was at least one case of infection and all studies reported on the number of patients who developed infection rather than the episodes of infection in each patient. Severity of infection was not reported and only 8 studies had information on how the infection was managed. Most infections were treated using antibiotics; debridement and implant removal was needed in some cases (Table 5).

**Table 5:** Management of Infections seen in the various studies (n = 8 studies)

Study	Antibiotics Only	Debridement	Implant Removal
Chapman,2003 (n = 1)	-	100%	-
Goddard,2010(n = 1) [34]	-	100%	-
Harrison, 2002 (n = 7) [35]	71.4%	14.3%	14.3%
Hoekman, 1991 (n = 12) [31]	58.3%	-	41.7%
Lehman,2001 (n = 8) [37]	-	12.5%	87.5%
Lofquist,1996 (n = 2) [38]	-	-	100%
Rodriguez,2007 (n = 1) [43]	-	-	100%
Silva,2005(n = 14) [44]	-	35.7%	64.3%

### The Effect of Prolonged Antibiotics in HIV Infected Patients

We only identified one study investigating the effect of prolonged antibiotics on post-operative infection after clean implant orthopaedic surgery in HIV patients when compared to antibiotics for up to 24 hours that had patients with post operative infection. This small good quality cohort study (n = 74) by Bahebeck *et al.* used cefuroxime 750 mg twice a day for 10 days for patients with CD4 counts less than 500 compared to 1500 mg of cefuroxime given at once during surgery for patients with CD4 counts above 500 [29]. The RR of infection in the patients receiving prolonged antibiotics compared to patients receiving antibiotics for up to 24 hours was 0.7 (95% CI 0.1–4.2). This indicated that patients with prolonged antibiotics had a reduced risk for infection but the study was not conclusive due to the wide confidence intervals. Another study used ARVs for some patients in addition to prophylactic antibiotics but had no patients with post operative surgical site infections [16].

## Discussion

In this systematic review and meta-analysis, we have selected studies in which a comparison between HIV patients and non-HIV patients undergo clean implant orthopaedic surgery. These studies were pooled to determine a possible increase risk in post-operative infection in HIV patients. From the overall meta-analysis, HIV infected patients were almost twice as likely to develop post-operative infection when compared to non-HIV patients undergoing clean implant orthopaedic surgery. This is likely due to decreasing resistance to infection due to dwindling numbers of immune cells [11]. This was also found in several subgroup analyses. However, the studies presented in these meta-analyses had several short comings. These included that most of the studies had small numbers of patients and hence the need for better designed larger studies, as well as it was unknown what the exact treatment status was of the included patients. As well, the percentage of patients (7.2%) in the cohort with post operative infection even in the non-HIV group were higher than the expected average of 2% after implant orthopaedic surgery, indicating a select group of enrolled patients [8]–[10].

Studies done in haemophiliac patients were all done in higher income countries, whilst studies conducted on non-haemophiliac patients coincided 100% with studies done in Africa. Though the cause of infections is multi-factorial, the difference in risk of infection between these two groups could be due to the differences in infection prevention strategies. The use of laminar flow theatres and surgeons wearing space-suits is common place in high income countries and could potentially reduce the possibility of infection by ensuring reduced contamination of the surgical site. Though patients with haemophilia have higher risks of infection due to frequent bleeding episodes into joints and the use of blood products, the majority of the studies were done on patients undergoing athroplasties where there are more enhanced measures to reduce infection like antibiotic loaded cement. Patients with haemophilia are generally not representative of the general population or of the HIV infected patient.

There were only four studies done in the African continent. Of these studies, the results indicate that there is an increased risk of infection in the HIV infected patients. This may be due to the less stringent infection prevention strategies in the operating theatres in less developed countries when compared to high income countries. However, these results are probably multi-factorial; for example the African studies had a higher number of trauma cases as opposed to athroplasties. In trauma the soft tissues are injured and hence more prone to infection as opposed to athroplasties where the soft tissues envelop is largely intact. The results of the studies done in Africa were heavily weighted by the largest study conducted by Jellis et al. [30]. It was difficult to determine the quality of this study as the method of determining patient HIV status and the follow up to determine infection was not adequately reported and hence we do not know if this may have led to an overestimation of the effect of HIV on post-operative infection. The rates of infection of both groups were also quite high, indicating selection bias. Also, the study by Hoekman et al. in Rwanda has also been criticized because they neglected to use any routine prophylactic antibiotics as suggested by current guidelines [31]. In a sensitivity analysis excluding the study by Jellis et al. due to poor quality (potential bias), the results changed and we found that the increased risk of postoperative infection due to HIV was no longer statistically significant.The conclusions in this subgroup are hence inconclusive.

In studies that reported on infection in the first 30 days which are classified as early infections, the results show no increase in risk for post-operative infection in HIV patients. However the data is very limited and hence larger, better designed studies are needed to address this question, specifically looking at both early infection and extended infections separately.

The consequences of infection can be grave. This is shown by the fact that in most patients who suffered infections required repeat surgery (debridement or implant removal). In the most severe cases, implant removal was required. In all cases this usually means prolonged hospital stay and long durations with intravenous antibiotics. In trauma surgery, the morbidity is less when compared to arthroplasty where implant removal means that the patient's joint may need to be fused if the infection cannot be controlled. The initial surgery is considered unsuccessful, the quality of life of the patient is reduced and hence there is reduced cost-effectiveness.

There was only one study conducted assessing the effect of enhanced antibiotic measures to reduce the risk of infection in HIV infected patients undergoing implant surgery. This study showed a small reduced risk of infection, but this finding was not significant. This showed that the study results were inconclusive as to the role of extended antibiotics in the reduction of the risk of postoperative infection in HIV patients. There is hence need for better designed studies with larger patient numbers that will help to answer this question. Due to the limited data available we were unable to do a subgroup analysis and determine the effect of CD4 counts and the use of ART. The different African countries have also different criteria for starting ART most of which are determined by funding available for ART programs. Though ART is currently available in many centers in Africa, most centers do not have access to the newer ARVs that have changed the management of HIV.

The funnel plot revealed symmetry in the distribution of the studies which means there may be little publication bias in this systematic review. There was however a paucity of studies not showing an increased risk of infection in HIV infected patients. This may have led to an overestimation of the risk of surgery in the HIV infected patients. This meta-analysis also reveals that there was little heterogeneity across studies even though the populations and settings were completely different. The studies included in the meta-analysis were mainly dealing with haemophilia patients in Europe and America. Though these studies may not represent the general population they indicate the patients in that region that are likely to require orthopaedic surgery and have HIV. In studies done in Africa, the patients included represented the general population and this makes the results of that subgroup analysis representative of the average patient in Africa.

Possible confounders across the studies in this meta-analysis include the effect of ART which was not reported in majority of the studies. ART is known to enhance the immunity of patients and may lead to possibly less infections. The varied use of antibiotics in type of drug, dosage and duration may be a potential confounder.

## Conclusion

From the results of the meta-analysis there seems to be a small increased risk of infection although the results are still inconclusive pending larger, better studies. There is currently no evidence for denying elective implant orthopaedic surgery to patients with HIV. Therefore surgeons should consider the individual patients needs very carefully and weigh the potential risk of operating against the quality of life of the patient. In elective surgery it may be necessary to first manage the HIV virus and attain viral load suppression and elevated CD4 levels before surgery.

To develop protocols for the treatment of HIV patients needing elective implant orthopaedic surgery, it is therefore imperative for large better conducted studies to determine the effect of declining CD4 counts and the use of ART and prolonged antibiotics on the risk of infection. There is also need for cohort studies to determine the risk of long term infection in implants that are left implanted for long periods of time as is the case for athroplasty implants. Even though the risk of post-operative infection is minimal and could potentially be reduced by ART treatment; surgeons may still decline to operate on this group of patients as there is also a concern that surgery in HIV patients could lead to a decline in the CD4 counts and possibly accelerate the progression to AIDS by additional stimulation of the immune system and surgical stress [32] This review does not look at this additional risk for the HIV patient.
